# Assessing the knowledge of emergency medical care practitioners in the Free State, South Africa, on aspects of pre-hospital management of psychiatric emergencies

**DOI:** 10.11604/pamj.2019.33.132.18426

**Published:** 2019-06-21

**Authors:** Jani Daniel Mothibi, Mpho Jama, Anthonio Oladele Adefuye

**Affiliations:** 1 Free State College of Emergency Care, Free State Department of Health, Free State, South Africa; 2 Division Health Sciences Education, Office of the Dean, Faculty of Health Sciences, University of the Free State, Free State, South Africa; 3 Division Student Learning and Development, Office of the Dean, Faculty of Health Sciences, University of the Free State, Free State, South Africa

**Keywords:** Emergency medical care practitioners, psychiatric emergencies, South Africa

## Abstract

**Introduction:**

Studies have reported that emergency medical care practitioners (EMCPs) encounter challenges when attending to psychiatric emergencies. The EMC provider's ability to understand, assess and manage psychiatric emergencies has been reported to be poor due to limited knowledge and insufficient training. In South Africa (SA), little is known about the knowledge of EMCPs on pre-hospital management of psychiatric emergencies. The objective of this study was to assess the knowledge of EMCPs working in the Free State province on aspects of pre-hospital management of psychiatric emergencies.

**Methods:**

This descriptive study used a questionnaire survey to obtain data on the knowledge of EMCPs on aspects of pre-hospital management of psychiatric emergencies.

**Results:**

Only 159 of the initial 192 questionnaires distributed were returned, giving a response rate of 82.8%. The majority (87.4%) of the participants reported inadequate knowledge of pre-hospital management of psychiatric emergencies. More than a third of the participants reported that they are not knowledgeable on how to assess a psychiatric patient (P < 0.01), 64.2% and 73.6% (P < 0.001 in both cases) could not perform mental status examination and lack the knowledge of crisis intervention skills for managing a psychiatric emergencies. The majority (76.7%; P < 0.001) of the participants are not conversant with the Mental Health Care Act 2002 (Act no. 17 of 2002). Finally, participants (94.3% and 86.8%, respectively; P < 0.001) agree that teaching and prior exposure to a psychiatric facility, as in work integrated learning, will empower EMC graduates with skills required to effectively manage psychiatric emergencies.

**Conclusion:**

EMC practitioners are often the first healthcare professionals arriving at any scene of medical emergencies including psychiatric emergencies. To avoid malpractices, which could be detrimental to patient's health, it is of utmost importance that EMCPs are well trained and equipped to manage any form of medical emergency including those involving psychiatric patients.

## Introduction

Globally, the burden of mental disorders continues to rise with significant impact on health, social, economic and human rights sectors [1]. Psychiatric emergencies (PEs) are acute onset of disturbance of behaviour, thought or mood of an individual which if untreated may lead to harm, either to the individual or to others [2]. Psychiatric emergency is a broad concept that consists of various disorders grouped into two major categories namely; acute excitement with psychomotor agitation and self-destructive or suicidal behaviour [3]. Psychiatric emergencies are often, but not always, caused by mental illness and about 60% of cases needing medical attention occur in non-psychiatric facilities [3]. According to Calzada and colleagues, acute agitation accounts for almost half of the total psychiatric emergencies in the pre-hospital setting [4]. Immediate treatment directed against these acute manifestations is needed, both to improve the patient's subjective symptoms and to prevent behaviour that could harm the patient or others [5].

In SA, the Life Esidimeni tragedy, that led to the death of 144 mental health care users and the torture of 1418 others [6], has raised important ethical and clinical issues [7]. This requires that healthcare professionals, including EMCPs are well trained on the ethical and clinical principles of managing psychiatric patients. EMC practitioners (EMCPs) are often the first healthcare professionals arriving at any scene of medical emergencies. An EMCP will routinely encounter patients with acute psychiatric disturbances in practice [8,9]. However, studies have reported that EMCPs encounter challenges when it comes to providing high-quality, safe and effective healthcare for the mentally ill [10,11]. It has been advocated that EMC personnel require mental health skills that will allow them recognise and manage mental illness in ways that will collaboratively add value to overall patient care [12]. At present, little is known about the knowledge of EMCPs in SA on pre-hospital management of psychiatric emergencies. Using a questionnaire survey, this study assessed the knowledge of EMCPs, working in the Free State province of SA, on aspects of pre-hospital management of psychiatric emergencies.

## Methods

This research was designed as a descriptive study that made use of a questionnaire survey.

***Questionnaire survey:*** the structured questionnaire used in this study was self-administered and was distributed manually (in hard copy) to the participants of this study. The questionnaire was compiled using factors identified during the literature review, which had been used by previous studies. Questions were adapted so that they were applicable to the context of the pre-hospital EMC environment. The questionnaire collected data in the following three sections; section A: biographical data; age, gender, qualification, district of operation, level of experience, EMC certification, and sector of practice, section B: knowledge survey questions; assessed participants knowledge on aspects of pre-hospital management of psychiatric emergencies. In this section, participants were asked to choose between “yes”, “no” or “unsure” in response to subject-specific, closed ended questions relating to the management of psychiatric emergencies in the pre-hospital setting. The open-ended questions requested that participants' supply a motivation for their response to the closed-ended question. The levels of knowledge assessed include; level 1: remember (K1) (The ability of the participants to recognise, remember and recall terms or concepts); and level 2: understand (K2) (The ability of the participants to be able to explain ideas or concepts) [13]. Section C: obtained participants' perceptions on the inclusion of teaching on pre-hospital management of psychiatric emergencies in the EMC curriculum in SA. Participants were requested to return completed questionnaire to the nearest emergency medical service (EMS) station in a box labelled for such purpose.

***Target population:*** the target population consisted of all EMC personnel working in the Free State provincial emergency medical services and private sector, who were registered (at the time of the study) with the Health Professions Council of South Africa (HPCSA).

***Sampling method and sample size:*** in this study, stratified random sampling was used to obtain a representative sample of 192 participants (10% of the entire population). The strata in this study were the different levels of EMC certification and the different sector of practice (government or private). The survey population consisted of individuals who were willing to participate in the study and complete the questionnaire.

***Pilot study:*** a pilot study was conducted to test the suitability of the study design and methods, the chosen data collection method and the overall structure of the questionnaire. The pilot study consisted of twelve EMC personnel at different levels of certification, and in different sector. The findings of the pilot study confirmed the feasibility of the main study, as the participants in the pilot study did not recommend changes to the structured questionnaire. The results of the pilot study were not included in the final results.

***Data collection and analysis:*** data collection was aided by EMS station managers and the drivers of the planned patient transport (PPT) system in the different regions, who assisted in both the dissemination and collection of the questionnaires. Quantitative data collated from the structured questionnaire was analysed quantitatively and results presented as frequencies and percentages. One-way ANOVA with Newman-Keuls multiple comparison post-test on Graph Pad Prism 4.0c (Graph Pad, San Diego, CA, USA) was used to determine significant differences between calculated mean percentages. Response to the open-ended questions are presented as participants verbatim quotes.

***Validity and reliability of the instrument:*** validity (Face validity, content validity, criterion validity, and construct validity) of the instrument used in this study was achieved by comparing the questionnaire elements with previous, similar studies and by conducting a pilot study. Furthermore, the questionnaire was subjected to review and approval by an evaluation committee, ethics committee and a senior biostatistician, all at the University of the Free State, Bloemfontein, South Africa. In order to achieve the reliability of the instrument, the closed ended questions were analysed with the use of Cronbach's alpha, within each subset of questions.

***Ethical considerations:*** approval to conduct the study was obtained from the Health Sciences Research Ethics Committee of the Faculty of Health Sciences at the University of the Free State (Ref. No. UFS-HSD2017/1184). Permission was also obtained from the Free State Department of health and a private EMS provider (name withheld).

## Results

Only 159 of the initial 192 questionnaires that was distributed were returned, giving a response rate of 82.8%. Of the participants, 78.0% (n = 124) were employed in the public sector (Free State Department of Health), while 19.5% (n = 31) were employed within the private sector. Four participants (2.5%) did not indicate the sector in which they were employed.

***Cronbach's alpha analysis of subset of questions:*** the knowledge survey questions subscale consisted of 12 items (α = .96), while the perception survey questions subscale consisted of 4 items (α = .88).

***Age of participants:*** majority (n = 47, 29.6%) of the participants were between 36-40 years, 22.6% (n = 36) were between 41-45 years, 22.0% (n = 35) between 31-35 years, 10.0% (n = 16) between 26-30 years, while only 5.7% (n = 9) of the participants were between 46-50 years. Four (2.5%) and 5 (3.1%) participants were between 20-25 years and older than 50 years, respectively. This data indicates the diversity of participants in relation to the age of EMC personnel. Seven (4.4%) participants did not indicate their age.

***Gender of participants:*** males made out 66.7% (n = 106) and females 32.0% (n = 51). Thus, suggesting a male predominance in the profession. Two (1.26%) of the 159 participants did not indicate their gender.

**Figure 1:**
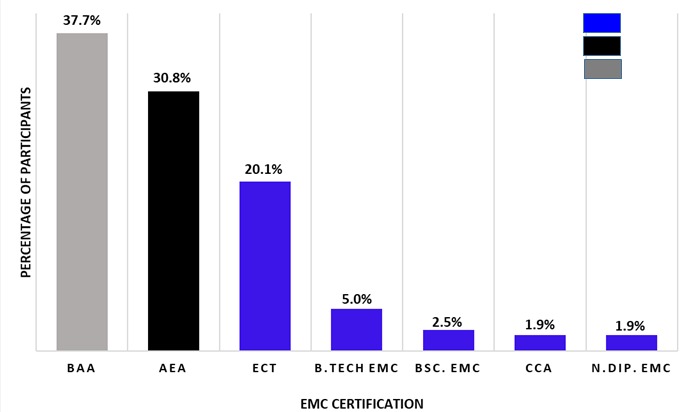
EMC certification of participants

***Qualification/level of training or EMC certification of participants:*** the majority (37.7%; n = 60) of the participants had basic life support (BLS) qualifications, 30.8% (n = 49) had intermediate life support (ILS) qualifications, and 31.4% (n = 50) had advanced life support (ALS) qualifications. Figure 1 shows the different certification represented in each cadre.

***Number of years post qualification:*** the majority (30.2%; n = 48) were 5-10 years post qualification, while 20.1% (n = 32) and 8.8% (n = 14) obtained their qualification 10-15 years and 15-20 years ago, respectively. A further 24.5% (n = 39) and 15.0% (n = 24) of the participants are 2-5 and less than 2 years post qualification, respectively. Only two (1.3%) of the participants obtained their qualification more than 20 years ago.

**Figure 2:**
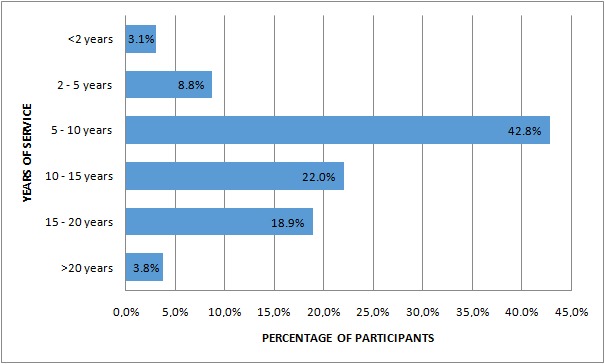
Duration of service as a pre-hospital emergency medical care practitioner (n = 158)

***Duration of service as pre-hospital EMC provider:*** the number of years that participants had been working as pre-hospital emergency medical care personnel is presented in Figure 2. The majority, that is, 42.8% (n = 68) of participants, indicated that they had been in service for between five and ten years. A further 22.0% (n = 35) had worked for 10-15 years, while only 3.1% (n = 5) and 3.8% (n = 6) had less than two years and greater than twenty years of service, respectively (Figure 2).

***Location of workplace:*** more than half (51.6%; n = 82) of the participants worked in urban settings (metropolitan/city); 28.3% (n = 45) worked in small towns; and only 18.2% (n = 29) worked in rural area. Two (1.26%) participants chose “other” as location of workplace. One participant did not answer this section.

***Level of employment:*** the majority 79.8% (n = 127) worked as operational staff, while those working as managerial staff account for 10.7% (n = 17). Nine (5.7%) participants were lecturers and six (3.8%) chose “other” as their level of employment.

***Prior experience in managing a psychiatric emergency:*** to establish if participants have had prior experience in managing psychiatric emergencies, participants were requested to respond to the question *“Have you attended to a psychiatric emergency before? ”* The majority (64.2%, n = 102) of the participants indicted “Yes” (P < 0.001), 26. 4% (n = 42) indicated “No”, while 9.4% (n = 15) were unsure.

***Knowledge of pre-hospital management of psychiatric emergencies:*** this section of the questionnaire focused on assessing participants' knowledge on aspects of pre-hospital management of psychiatric emergencies.

***Participants' self-appraisal of their knowledge on pre-hospital management of psychiatric emergencies:*** participants were asked to do a self-appraisal of their knowledge on pre-hospital management of psychiatric emergencies by answering “Yes or No or Unsure” to the question *“Do you feel confident with the level of your knowledge regarding pre-hospital management of psychiatric emergencies? ”* Result obtained revealed that the majority (87.4%, n = 139) indicated “No”, while only 2.5% (n = 4) indicated “Yes”. Fourteen (8.8%) participants were “Unsure” whether their knowledge of pre-hospital management of psychiatric emergencies is adequate.

***Knowledge on the different types of psychiatric emergencies:*** here, participants were asked to respond to the question; *"Do you know the different types of psychiatric emergencies?"*. Only 22.6% (n = 36) of the participants indicated that they know the different types of psychiatric emergencies by indicating “Yes” (cf. quotes #1-#3), 47.8% (n = 76) indicated that they do not know by choosing “No”, while 29.6% (n = 47) were unsure. *#1 “anxiety disorder; personality disorders; violent disorder; substance abuse”, #2 “delirium/dementia; psychosis; attempted suicide or deliberate self-harm; alcohol or drug overdose; withdrawal symptoms of drug dependence; panic, violence or excitement”, #3 “suicide attempts, violent behaviour, psychosis, personality disorders, substance abuse and intoxication”.*

***Knowledge on how to approach an individual with psychiatric emergency:*** only 33.3 % (n = 53) of the participants indicated that they are knowledgeable on how to approach an individual with psychiatric emergency (cf. quotes #4 and #5), 38.3% (n = 61) indicated that they are not knowledgeable (cf. quotes #6 and #7), and 27.7% (n = 44) are unsure of how to approach such case (cf. quote #8). *#4 “approach patient in a calm manner to gain their trust and see if the patient is willing to work with you after gaining their trust”, #5 “try to calm and reassure patient. Take the family member with when talking with the patient”, #6 “I did not receive any structured formal/informal training, nor any exposure during any training period”, #7 “no I have zero skills to handle them so am even scared to approach. They might harm me”, #8 “every psychiatric pt is different, so I would say it differs”.*

***Knowledge on how to assess an individual with psychiatric emergency:*** the majority (46.5%, n = 74) of the participants indicated that they are not knowledgeable on how to assess an individual with psychiatric emergency (P < 0.01) (cf. quotes #9-#11), only 19.5% (n = 31) indicated that they know how to assess such patient (cf. quotes #12 and #13), while 34.0% (n = 54) indicated that they are unsure (cf. quote #14). *#9 “usually we contact SAPS for assistance”, #10 “no education on psychiatric patient”, #11 “no proper guide lines, how to assess specifically psychiatric patient like other cases/emergencies”, #12 “obtain hx. Talk to the pt ask relevant questions”, #13 “assess mental status; patient behaviour ”, #14 “I don't know the exact signs of a compute psychiatric patient”.*

**Knowledge on mental status examination/assessment protocol for psychiatric patients:** only 5.7% (n = 9) of the participants reported that they have knowledge of a mental status examination/assessment protocol for psychiatric patients (cf. quotes #15 and #16). The majority (64.2%, n = 102) of the participants reported lack of knowledge of a mental status examination/assessment protocol for psychiatric patients (P < 0.001), while 29.6% (n = 47) were unsure whether they possess this knowledge. #15 “I am aware of the MSE chart which provides many criterion to be taken into count”, #16 “mini mental state examination (used in SA) DSM5”.

**Knowledge on crisis intervention skill for managing psychiatric emergencies:** when asked to indicate whether they are knowledgeable on crisis intervention skill for managing psychiatric emergencies, the majority (73.6%, n = 117) of the participants indicate lack of knowledge i.e. “No” (cf. quotes #17 and #18) (P < 0.001), 23.3% (n = 37) were not sure if they possess such knowledge (cf. quote #19), while only 1.9% (n = 3) reported that they are knowledgeable on crisis intervention skill for managing psychiatric emergencies (cf. quote #20). #17 “no formal/informal training on the subject”, #18 “no skills that is why when I approach them and realize they are insane I call the police”, #19 “I have never been taught the skills for crisis intervention in managing psychiatric patients, and I am not sure if there is any skills required regarding psychiatric patients”, #20 “stay calm and be careful when handling such patients call for assistance if patient becomes violent eg. Higher qualified person or police. If patient present with medical condition injury treat the emergency as per protocol”.

***Knowledge of the Mental Health Care Act 2002 (Act no. 17 of 2002) of the Republic of South Africa:*** here participants were asked whether they are conversant with the Mental Health Care Act 2002 (Act no. 17 of 2002) of the Republic of South Africa. Of the respondents, only 10.0% (n = 16) reported that they were knowledgeable on the Act, by indicating “Yes”, the majority (76.7%, n = 122; P < 0.001) reported lack of knowledge of the Act, while 11.9% (n = 19) were unsure. Two participants did not respond to this question.

### Participants' perceptions on the inclusion of teaching on pre-hospital management of psychiatric emergencies in the EMC curriculum

Participants were asked to indicate the extent to which they “Agree” or “Disagree” with the statement in Table 1.

**Table 1:** Participants’ perceptions on the inclusion of teaching on pre-hospital management of psychiatric emergencies in the EMC curriculum

Statements	Agree n (%)	Disagree n (%)	No response n (%)
Do you think you are likely to attend to a psychiatric call?	117 (73.5)***	40 (25.2)	2 (1.3)
Do you think prior exposure to psychiatric facilities will empower EMC graduates with skill to manage psychiatric patients?	138 (86.8)***	18 (11.3)	3 (1.9)
Do you think it is necessary to include teaching on pre-hospital management of psychiatric emergencies in the EMC curriculum?	150 (94.3)***	6 (3.8)	3 (1.9)

*** P < 0.001

## Discussion

According to the World Health Organization (WHO) report on mental disorders, around 450 million people worldwide currently suffer from mental disorders and one in four people in the world will be affected by mental or neurological disorders at some point in their lives [14]. Accompanying this high prevalence are reports confirming the increase in the frequency of psychiatric emergencies presenting to emergency departments and emergency medical services globally [15-19]. Pre-hospital emergency care is an essential part of the continuum of emergency health care provided by emergency medical services (EMS) responders [20]. EMCPs are the initial health care providers at the scene of any medical emergency and are often the first to evaluate the nature of the emergency, determine the need for medical resources, initiate management and provide medical transport for the sick and injured [20,21]. However, it has been reported that the EMC provider's ability to understand, assess and manage psychiatric emergencies remains poor [22], due to limited knowledge and insufficient training [18]. Moreover no known study has investigated the knowledge of EMCPs in SA on aspects of pre-hospital management of psychiatric emergencies. This present study assessed the knowledge of EMCPs, in the Free State province of SA, on aspects of managing psychiatric emergencies in the pre-hospital setting.

The South African health care sector has been described as a two-tiered system divided into the government funded public healthcare sector and the private healthcare sector, where citizens must purchase their own private medical insurance in order to be treated at a private healthcare facility [23]. Similarly, employment of health professionals is either through government institutions (public health sector) or self-employment/employment by cooperate bodies in the private sector [24]. Majority (78.0%) of the participants of this study indicated that they are employed in the public sector. Thus, suggesting that majority of EMCPs within the Free State province are employees of the Free State Department of Health. This is contrary to studies that report a preference for the private health sector by health professionals in South Africa, due to poor working conditions in the public health sector [25,26]. However, the disparity in public vs private employee reported herein is justified since only one private employer gave permission for its practitioners to participate in the study. Other private employers approached declined request to participate in the study.

Traditionally, the emergency medical care sector is considered a male-dominated field [27]. A male dominance of 66.7% compared to 32% female presented herein corroborates findings in published literature [28-30] and thus suggest that the EMC profession is still dominated by male practitioners in the Free State province. This situation is not unique to the Free State province as many EMS across the country are still lacking female practitioners and concerns about gender bias in the profession has been reported [28,31]. The predominance of male over female in the EMC profession is partly because prior to the establishment of the EMC as a profession, pre-hospital emergency medical services were initially offered by the fire departments, a traditionally male-dominated field, in most communities [27,28]. Findings presented by this study suggest that EMCPs within the Free State province are relatively young (median age group 36-40 years) with majority having between 5 and 10 years of working experience in the EMC sector (Figure 2). This corroborates similar findings by Butler (2015) [31].The certification of an EMC practitioner can be categorized in to three main cadres namely; Basic Life Support (BLS), Intermediate Life Support (ILS) and Advanced Life Support (ALS) [32]. From this study it can be said that only a small number of EMC practitioners in the Free State province are in possession of ALS certification while, the majority are in possession of a BLS or ILS certification (Figure 1). Certification for BLS can be obtained after undergoing a 4-6 week course, while the ILS certification is obtained after attending a 4 months course that builds on the foundation laid during the BLS training (BAA course) [32]. The BLS practitioner (BAA), is capable of doing the basics including (but not limited to) simple airway management, extrication and splinting of fractures [33], but lack training in psychiatry emergency care. This may have an impact on the scope of pre-hospital emergency care delivered by such personnel and ultimately patient outcome [34].

Despite 64.2% of the participants indicating that they have attended to a psychiatric emergency case before, the majority (87.4%) of the participants across the cadres reported that they are not confident of their knowledge on pre-hospital management of psychiatric emergencies. Thus, suggesting that majority of the patients attended to by these 102 (64.2%) EMCPs were poorly managed. While the EMCP is not expected to diagnose patients with psychiatric emergencies, it is vital that the practitioner has a thorough understanding of the presentation and management of such patients in order to provide safe and expedient transport to definitive care [8]. In this presented study, the majority (47.8%) of the participants indicated that they are not knowledgeable on the different types of psychiatric emergencies. In cases involving suicidal ideations, the EMCP is expected to approach the individual with a degree of understanding of their turmoil and speak in a calm, supportive manner [22]. Findings by this study reveal that more than a third of the participants indicated that they are not knowledgeable on how to approach an individual with psychiatric emergency. This was attributed to lack of training or exposure (cf. quotes #6 and #7).

Mental State/Status Examination (MSE) is part of every mental health assessment. The MSE is used to gain an in-depth understanding of the patient's psychological functioning at a particular point in time in order to direct care appropriately [35]. In cases involving suicidal ideations, the EMCP is required to perform a thorough assessment to determine if there is enough evidence to seek help in obtaining an involuntary commitment/transport should the patient be unwilling to seek help [22]. Findings by this study reveal that only 5.7% of the participants indicated that they are knowledgeable on a MSE protocol (cf. quotes #15 and #16), while the majority (64.2%) reported lack of knowledge. In addition, only 19.5% of the EMC practitioners in this study indicated that they are knowledgeable on how to assess an individual with psychiatric emergency (cf. quotes #12 and #13), while the majority (46.5%) reported lack of knowledge, which they ascribe to no education and the absence of proper guidelines (cf. quotes #10 and #11). From these findings, it can be said that most EMC practitioners within the Free State province and indeed SA are not skilled in examining/assessing a case of psychiatric emergency in the pre-hospital setting. During psychotic relapse, sufferers may experience a sudden exacerbation of acute symptoms which could be life threatening to the patient or others. Initiating crisis intervention at this stage is crucial as it brings much needed relief for both the patient and their carer and can help prevent further deterioration [36]. Studies have reported that early crisis intervention, with immediate reduction of psychotic symptoms is beneficial for the long-term prognosis of this illness [37,38]. In this present study, the majority (73.6%) of the participants indicated that they are not knowledgeable on crisis intervention skill for managing a psychiatric patient (cf. quotes #17 and #18). This could lead to poorer long-term prognosis and poor patient outcome.

In SA, like in many other parts of the world, patient care takes place within a legal framework consisting of legislations, Acts, policies and laws to protect patient's rights. The Mental Health Care Act 2002 (Act no. 17 of 2002), ushered in a new era for South African psychiatry by replacing the Mental Health Act of 1973 [39]. A requirement of the Act is that mental health care user be treated in the least restrictive manner possible and ultimately with the least discomfort and inconvenience [40]. Findings presented herein reveal that the majority (76.7%) of the EMCPs who participated in this study indicated that they are not knowledgeable on the provisions of the Act. Thus, suggesting that a good majority of EMCPs in the Free State province and indeed SA are not conversant with the provisions within the Mental Health Care Act. This may lead to varying malpractices that may be detrimental to patient's health. When asked about their opinion on the inclusion of teaching on pre-hospital management of psychiatric emergencies in the EMC curriculum, the majority (94.3% and 86.8%, respectively) of the EMCPs agree that teaching and prior exposure to a psychiatric facility, as in work integrated learning, will empower EMC graduates with skills to effectively manage psychiatric emergencies.

***Limitations of the study:*** limitations of this study include; selective answering of the questions in the questionnaire by the participants i.e. in some cases, respondents only answered the first part of a question and not the follow up second part of the question, for example giving reasons for their answer, unequal representation as the majority of the participants of this study are only BLS and ILS certified. While, this is recognised as a limitation, the overall goal of the study was to assess the knowledge EMC practitioners on pre-hospital management of psychiatric emergencies.

## Conclusion

EMCPs are often the first healthcare professionals arriving at any scene of medical emergencies including psychiatric emergencies. It is therefore of utmost importance that EMCPs are well trained and equipped to manage any form of medical emergency including those involving psychiatric patients. Enhancing their knowledge and skill in pre-hospital management of psychiatric emergencies will ensure that adequate and comprehensive pre-hospital emergency care is given to this vulnerable group of patients. Resources such as management guidelines and or structured protocol on how to manage psychiatric emergencies in the pre-hospital environment may be useful. Furthermore, organising continuing professional development (CPD) course on management of psychiatric emergencies in the pre-hospital setting can be useful in training EMCPs already in practice.

### What is known about this topic

- It has been established that there is a poorly coordinated emergency health care system in some parts of Africa;
- Prior studies in Germany and Australia have documented the poor knowledge of EMCPs in the management of psychiatric emergencies, but no known study has investigated the knowledge of EMCPs in SA on the pre-hospital management of psychiatric emergencies.

### What this study adds

- This study found that some EMCPs in the Free State province of SA are not knowledgeable about aspect of pre-hospital management of psychiatric emergencies and are not conversant with the provisions of Mental Health Care Act 2002 (Act no. 17 of 2002) of SA;
- This study adds, furthermore, that poor or lack of knowledge by the EMCPs could lead to varying malpractices that may be to the detriment of the patients;
- To address this knowledge gap and prevent malpractice, this study offers implementable recommendations (providing management guidelines and or structured protocol, organising CPD course on management of psychiatric emergencies in the pre-hospital setting, and inclusion of training on pre-hospital management of psychiatric emergencies in the EMC curriculum in SA) to enhance the knowledge of EMCPs personnel in the province and indeed SA on pre-hospital management of psychiatric emergencies.

## Competing interests

The authors declare no competing interests.
